# Magnitude and Shape of the Forces Applied on the Foot Rest and Paddle by Elite Kayakers

**DOI:** 10.3390/s22041612

**Published:** 2022-02-18

**Authors:** Pedro Bonito, Miguel Sousa, Fernando José Ferreira, Jorge Fonseca Justo, Beatriz Branquinho Gomes

**Affiliations:** 1 Faculty of Sport Sciences and Physical Education, University of Coimbra, 3040 Coimbra, Portugal; pedro_a_bonito@hotmail.com; 2 Department of Mechanical Engineering, School of Engineering, Polytechnic of Porto, ISEP/IPP, 4200 Porto, Portugal; miguelfbsousa@yahoo.com (M.S.); fjf@isep.ipp.pt (F.J.F.); jfj@isep.ipp.pt (J.F.J.); 3 Research Unit for Sport and Physical Activity—CIDAF (uid/dtp/042143/2020), University of Coimbra, 3040 Coimbra, Portugal

**Keywords:** kayaking, kinetics, lower limbs, load cells, foot strap, foot rest, force measurements

## Abstract

The study aimed to investigate the magnitude and shape of the forces applied on the foot rest, foot strap, and paddle. Thirteen elite male kayakers participated in this study and performed a 2-min test simulating 500 m race pace in a kayak ergometer. Forces applied by the kayakers on the paddle, foot rest, and foot strap were measured with load cells and recorded by an electronic measuring system. The magnitude of the peak forces applied on the foot rest (left: 543.27 ± 85.93; right: 524.39 ± 88.36) approximately doubled the ones applied on the paddle (left: 236.37 ± 19.32; right: 243.92 ± 28.89). The forces on the foot strap were similar in magnitude to the paddle forces (left: 240.09 ± 74.92; right: 231.05 ± 52.01). A positive correlation was found between the peak forces applied on the foot rest and paddle on the same side (*p* < 0.001). When comparing the best and worst kayakers’ performance, the best showed greater forces magnitudes and synchronization of the peak forces. Analyses of the force–time curves, including not only the forces applied by the kayaker on the paddle but also the ones applied on the foot rest and strap, should be considered relevant in terms of technique analyses.

## 1. Introduction

In sprint kayaking, the athlete produces force to move the kayak forward, and these forces are transmitted to the water by the paddle blade and to the kayak by the foot rest, foot strap, and seat [1]. The introduction of the ”wing” blade originated changes in the paddling technique with a tendency for a lateral paddle path during the water phase of the stroke, which increased the contribution of the trunk rotation and a great lower limbs movement to force development and performance [1]. The greater lower limb action and rotation of the pelvic girdle have been associated with the performance of more experienced/high-level kayakers [2,3,4]. When comparing performances involving restricted and unrestricted pelvic girdle and lower limbs movement on a kayak ergometer, unrestricted movements were associated with better blade velocities (+0.2 to 0.4 m∙s^−1^), higher paddling impulse (+3.5 N∙s), significant increases in strength throughout the paddle cycle, decrease in mechanical work (−4%), better propulsion (+6%), and consequently an increase in the performance (+6%) [5]. These results were confirmed in an on-water kayaking study, also comparing restricted and unrestricted, and concluded that the restriction of lower limbs leads to a significant reduction of the kayak velocity (−16%) and a decrease of the bilateral mean stroke force (−21%), however, in terms of the time pattern of the occurrence of the peak force on the foot rest and foot strap, there were no significant changes [1]. Futhermore, the impulse of the push forces on the foot rest over 10 s showed the highest correlation to maximum kayak velocity [6].

A small number of studies have analyzed kayaking kinetics and its relationship with performance on a kayak ergometer [2] and on-water situation [7,8] and were more centered on the forces applied on the paddle. In these studies, the best performances were associated with paddle force–time curves where the kayakers reached peak force quickly and tended to maintain a force near peak values until the exit of the paddle from the water. Gomes and colleagues [7] reported that the mean kayak velocity had a higher correlation with mean paddle force compared with peak force, therefore, when the area under the force–time curve increased and the stroke frequency was maintained, the speed of the kayak increased. They also reported that the elite paddlers’ performance was characterized by shorter water phase duration, higher handgrip loading, higher blade force, and impulse compared to a recreational paddler [7,8].

With regard to the study of the forces applied on the paddle, but also on the foot rest and strap, Tornberg and colleagues [9] analyzed these forces in a pilot study and reported that high-level kayakers produced the same forces on the paddle compared to the low level ones, but in relation to the forces applied on the foot rest, the high level ones produced 3 to 26 times more force. A subsequent study [10], which also intended to analyze these forces but on water, showed that the forces applied on the foot rest began to be applied before the paddle forces on each stroke. However, this study could not report in detail the magnitude of the forces applied on the foot rest or strap, being only valid for the timing of the different movements [10].

A few studies [1,6,9] have been dedicated to the analyses of the forces applied on the foot rest and foot strap, and none have analyzed the magnitude and shape of the force–time curves of compression (push foot rest forces), tension (pull foot strap forces), and paddle, as well as the synchronization between these forces in elite kayakers. Therefore, the goals of the present study were to describe the magnitude and shape of the force–time curves of compression and tension applied on the foot rest and foot strap, respectively, by studying elite kayakers and their performance on a kayak ergometer, and simultaneously, to analyze the synchronization and timing of these forces with the paddle force–time curves.

## 2. Analysis of Interaction between the Kayaker, Paddle, Kayak and Water

**Figure 1:**
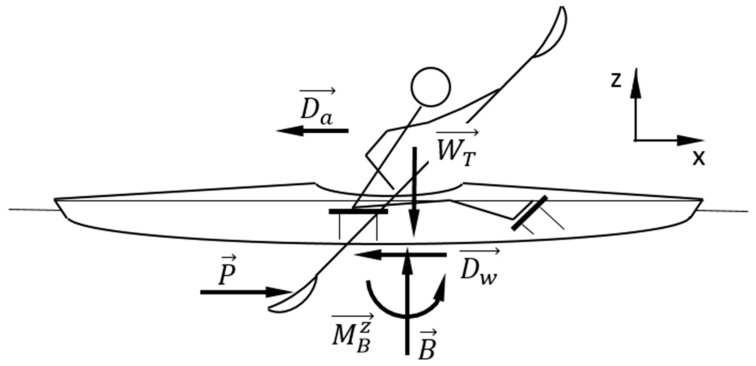
Kayaker/kayak/paddle free body diagram.

To improve the kayaker’s performance, it is essential to understand the mechanics of interaction forces between the kayaker, paddle, kayak, and water. To assess the efficiency of the paddling movement, it is vital to quantify all the actions (forces and moments) that the kayaker exerts during this process. Figure 1 shows all the actions on these three bodies, where $\vec{W_{T}}$ is the total weight of the three bodies; $\vec{P}$, paddle propulsion force; $\vec{D_{w}}$, water drag force; $\vec{D_{a}}$, air drag force; $\vec{B}$, buoyant force; $\vec{M_{B}^{z}}$, moment caused by water distributed on the hull. Considering their small magnitude, $\vec{D_{a}}$ and $\vec{M_{B}^{z}}$ were neglected.

**Figure 2:**
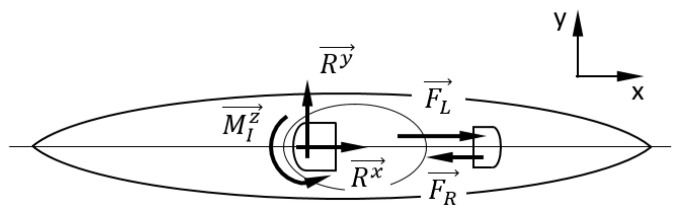
Kayaker/kayak interaction diagram.

This study focused on the following forces developed by the kayaker: $\vec{P}$, paddle propulsion force; and foot rest forces, $\vec{F_{L}}$ and $\vec{F_{R}}$. The interaction forces between kayaker and kayak in the Oxy plane are shown in Figure 2. The kayak seat receives the main weight of the kayaker, whereas the foot rest and foot strap receive the main forces developed by the kayaker legs/feet. The foot rest force applied by one foot, combined with the foot strap force applied by the other foot, creates a moment along the *z*-axis that makes it possible for the kayaker to rotate its pelvic girdle for paddling left and right and counterbalances the moment created by the paddle propulsion force, $\vec{P}$ (Figure 2).

## 3. Materials and Methods

### 3.1. Instrumentation and Devices

**Figure 3:**
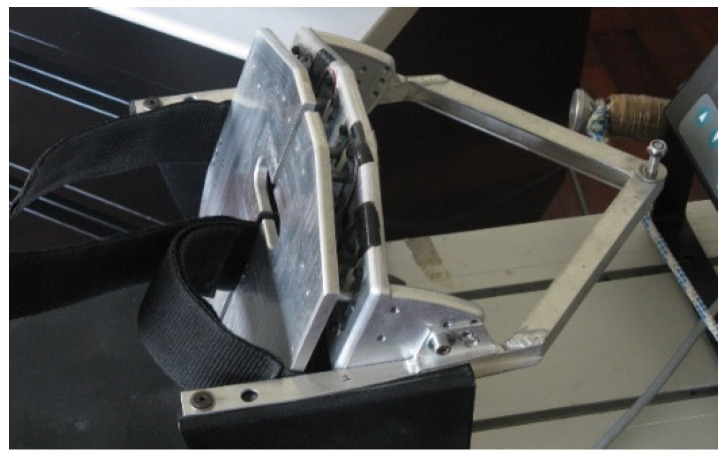
Custom-built foot rest, that measure push and pull forces over the foot rest and foot strap, respectively.

**Figure 4:**
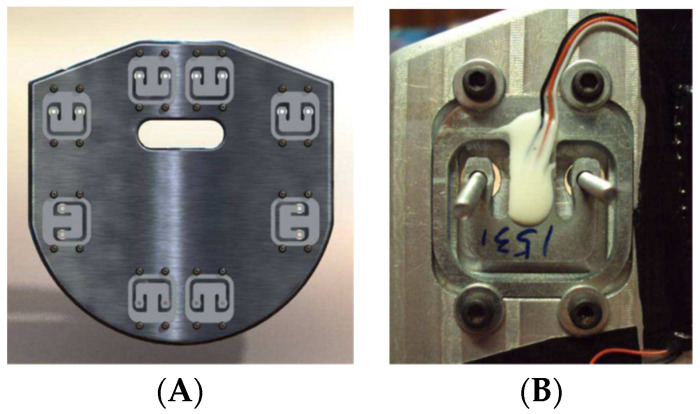
(**A**) View of the foot rest’s interior with four load cells on each side, (**B**) the detail of one load cell.

The foot rest/strap was custom-designed and built at the ISEP/IPP, School of Engineering. This foot rest/strap (Figure 3) has a structure similar to the shape of the original foot rest and is divided into two parts, each one having four double strain gauge load cells (Figure 4), allowing measurement of the left and right forces independently. These load cells work in both directions, measuring push and pull forces (compression and tension forces, respectively). The position of the four load cells on each side was chosen so they would work similarly to the supports of a scale. The load cells are connected in a full Wheatstone bridge with 8 active strain gauges, resulting in an instrumented foot rest that is insensible to the location of the applied force.

**Figure 5:**
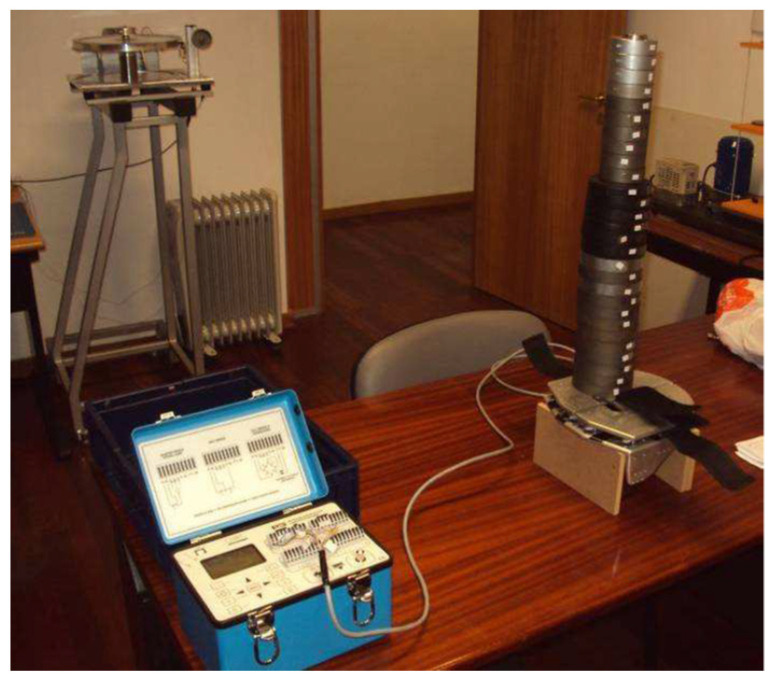
Foot rest calibration procedure.

Each side of the foot rest was calibrated in the laboratory using calibrated weights and a Micro-Measurements VISHAY P3 signal conditioner (Figure 5).

**Figure 6:**
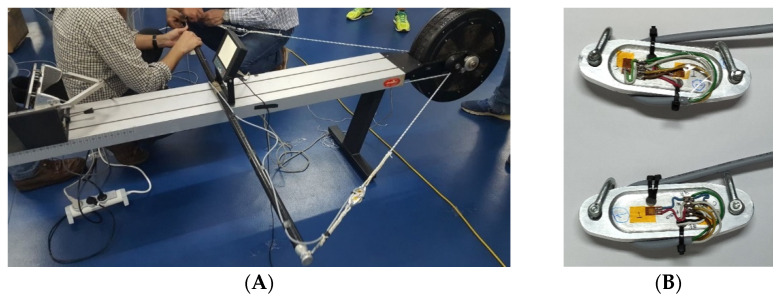
(**A**) Kayak ergometer with instrumented foot rest and paddle ropes, (**B**) custom design load cells built for measuring kayak ergometer paddle ropes forces.

The ergometer paddle drive ropes were instrumented with load cells (Figure 6) custom-designed and built for this application. These load cells were designed to work in tension, their mechanical behavior was assessed using finite element software. They were manufactured from an aluminum alloy 5083 piece in a CNC machine at ISEP/IPP. Each one has 4 strain gauges in a full Wheatstone bridge configuration. Both were calibrated at ISEP/IPP laboratory using calibrated weights and the signal conditioner VISHAY P3.

### 3.2. Participants

The study was conducted with thirteen elite male kayakers (5 senior and 8 under-23 athletes), age 23.3 ± 4.9 years, height 179.0 ± 5.7 cm, and a body mass of 79.70 ± 5.70 kg. The senior kayakers had international participations, including World Championships and Olympic Games. The under-23 kayakers had participated in International junior and under-23 competitions in the last two years. The participants were selected considering the performance times in the 500 m of the Sprint National Cup and participation in World Cups and World Championship. Subjects were fully informed of the nature of the investigation and provided written informed consent before data acquisition. The study was approved by the Institutional Review Board performed according to the Declaration of Helsinki.

### 3.3. Procedures

To observe and describe the magnitude and shape of the forces applied on the foot rest, foot strap, and paddle, a kayak ergometer (Dansprint Pro, Denmark) [11] was instrumented and data from the forces applied on the foot rest and strap, compression and tension, were synchronized with data of the tension on the paddle drive ropes. Kayak ergometer has been considered a valid tool for physiological testing [12]. In terms of biomechanical demands, these probably do not fully replicate [13], however, it has been shown that there are no significant differences in terms of paddle force variables—peak force, time to reach peak force, and impulse [14]. The kayakers that participated in the present study were familiar with the ergometer, considering that they are often used for training, mainly in the winter season, and that their training control tests are usually performed on a kayak ergometer. The drag of the ergometer was adjusted considering the kayaker’s body mass as recommended by the Dansprint to simulate on-water paddling drag.

A 2-min test race pace was performed on the kayak ergometer simulating 500 m sprint race performance. The warm-up consisted of a high-intensity interval exercise for kayak ergometer adapted from Bishop and colleagues [15]. At the end of the warm-up, the athletes rested passively for 5 min and then performed the test. After the 2-min test they performed a 10-min active recovery on a static bike at 60 rpm.

### 3.4. Kinetic Data Collection

Data processing and storage was performed using a PC and a signal conditioner system (HBM^®^ Spider8, Darmstadt, Germany). Data was recorded at 100 Hz sample rate. The system synchronously recorded data from all channels (left and right foot rest, left and right paddle ropes), as the different channels were connected to the signal conditioner using the same time base for all channels.

### 3.5. Data Analysis 

The data was processed by MATLAB R2016a (The MathWorks Inc., Natick, MA, USA) and analyzed using an in-house developed routine. The routine automatically detected the strokes by analyzing both the force data from the load cells on the paddle rope (left and right) synchronized with the foot rest/strap load cells. Paddle (P) and foot rest/strap forces (expressed in newtons) were analyzed for each side (l, left and r, right). On the foot rest/strap the compression (C) forces represented positive values of the curve in the direction of kayak travel considering the pull forces applied to the foot rest, and the tension (T) forces represented negative values of the curve, in the opposite direction by pulling the foot strap. The MATLAB routine identified the compression stroke onset when the force applied on the foot rest/strap crossed from negative to positive, and its end, when again the force went from positive to negative. The opposite was used for the tension forces, so that the onset occurred when the force applied on the foot rest/strap went from positive to negative and its end when the values went positive again. For the cable rope force analysis, the onset and end of the paddle stroke in the left and right sides was defined when, for two consecutive values, the data was above or below 10 N, respectively.

**Figure 7:**
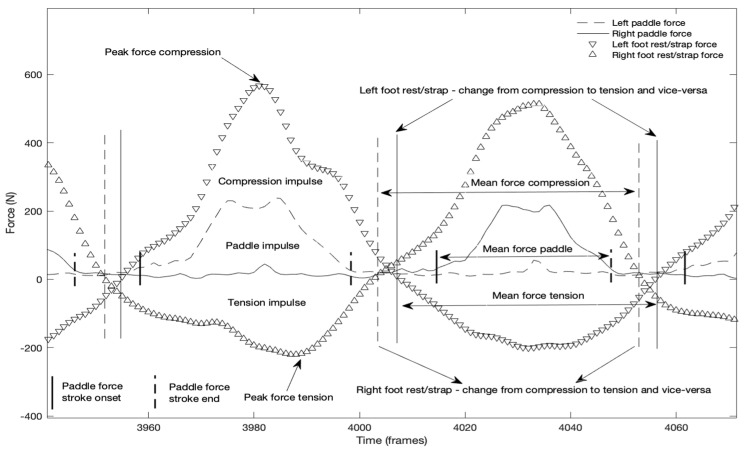
Example of two complete paddle strokes (left and right) presenting the paddle, foot rest, and foot strap force curves synchronized, and the main kinetic variables considered for analysis.

In each of the force curves (Figure 7), and considering all the paddle strokes, the peak force (PF), mean force (MF), and impulse (I) were computed. The PF represented the maximum value reached, the MF the average value of the forces and impulse the area below the force curve (force–time integral). The foot rest and strap variables that were considered for analysis were:

(1) left and right peak force compression (CPF) over the foot rest, and left and right peak force tension (TPF) over the foot strap;
(2) left and right compression mean force (CMF) over the foot rest, and left and right tension mean force (TMF) over the foot strap; and
(3) left and right compression impulse (CI) over the foot rest, and left and right tension impulse (TI) over the foot strap.

The previous variables (PF, MF, and I) were also studied for the paddle force curves: (1) left and right peak paddle force; (2) left and right mean paddle force; and (3) left and right paddle impulse. 

The mean force/peak force ratio (MF/PF R) was computed for compression and tension curves, as well as for paddle force curves, and reflects the force profile, where a rectangular shape is represented by 100% and closer to 50% is a tendency for a triangular shape [16]. 

Time variables were also computed for each stroke: (1) the duration in time of the compression and tension forces, as well as paddle force application in each side; and (2) the time to peak force of the compression and tension forces applied to the foot rest/strap and on the paddle. 

The performances with the higher and lower mean power reached on the kayak ergometer (given by the kayak ergometer software) during the 2-min test were analyzed in detail. When comparing the best with worst performance, the variables mentioned above were analyzed in absolute and also normalized considering the kayakers’ weight.

### 3.6. Statistics

All statistical analysis was conducted using SPSS v25.0 (SPSS Inc., Chicago, IL, USA). Descriptive statistics included mean and standard deviation. The data were checked for distribution normality and homoscedasticity with shapiro–Wilks and Levene tests, respectively. T-student for paired samples was used to compare differences within each variable (foot rest/strap and paddle) between left and right sides. Pearson correlation test was computed for the analysis of the correlations between these variables and also to correlate left and right sides forces. A correlation matrix was designed on MATLAB R2016a (The MathWorks Inc., Natick, MA, USA). The confidence interval was set at 95%. For the analysis of the differences between the best and worst performances, the difference in percentage for each variable was computed—Dif. % = ((BF − WP)/((BF + WP)/2) × 100), where BF is the result in each variable of the kayaker’s best performance and WP is the kayaker with the worst performance.

## 4. Results

### 4.1. Analysis of the Magnitude and Shape of the Force Applied on the Foot Rest/Strap and Paddle

The mean power generated by the kayaker on the kayak ergometer was set as the performance indicator (Table 1). Table 2 presents the magnitude values of the forces applied on the foot rest and strap, time variables, and the profile of the force curves, considering the ratio mean force/peak force. No differences were observed, when comparing the mean values of the forces applied on the left and right foot rest and foot strap. The magnitude of the peak forces of compression over the foot rest more than doubled the tension peak forces applied over the foot strap; the same was observed for impulse. The duration of force application was higher for compression than tension. Since left pull (compression) occurs simultaneously with right push (tension) over the foot rest/strap, and vice-versa, the data showed a similar mean difference in duration (0.07 s). The force profile (MF/PF R) for the compression forces tended towards a triangular shape (≈53%) and for tension forces tended more towards a rectangular shape (≈63%).

Data from the analyses of the forces applied on the paddle are presented in Table 3. There were no statistical differences between the magnitude of the forces applied on the left and right paddle sides (*p* > 0.05).

**Figure 8:**
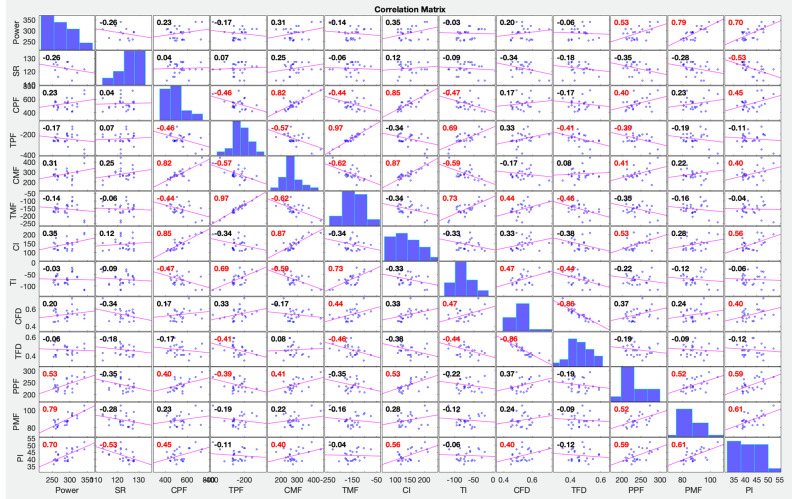
The correlation matrix with correlation coefficients between power and force variables of the paddle, foot rest and foot strap, combining left and right of 13 kayakers. Red correlation coefficients indicate strong correlation, the tendency line shows if its positive or negative. SR—stroke rate; CPF—compression peak force; TPF—tension peak force; CMF—compression mean force; TMF—Tension mean force; CI—compression impulse; TI—Tension impulse; CFD—compression force duration; TFD—tension force duration; PPF—paddle peak force; PMF—paddle mean force; PI—paddle impulse.

The performance (mean power on the kayak ergometer) presented a positive correlation with the paddle mean force (r = 0.792, *p* < 0.001) and paddle impulse (r = 0.701, *p* < 0.001), respectively (Figure 8). The stroke rate presented a negative correlation with paddle impulse (r = −0.530, *p* < 0.01). Also, a correlation was found between the variables in analyses of the paddle stroke, when analysing left and right sides (peak force—r = 0.786, *p* < 0.01; mean force—r = 0.670, *p* < 0.05; impulse—r = 0.648, *p* < 0.05). 

The compression peak force correlated negatively with the tension peak force applied on the opposite side of the foot rest/strap (r = −0.461, *p* < 0.05) and the same was observed for the analysis of the mean force values, where compression mean force correlated negatively with the tension mean force applied on the opposite side of the foot rest/strap (r = −0.613, *p* < 0.001). Left and right compression mean forces were also correlated (r = 0.874, *p* < 0.01). When variables related to the forces applied to the foot rest and strap were correlated with paddle forces, paddle peak force showed a positive correlation with the compression peak force (r = 0.400, *p* < 0.05) and with the compression impulse (r = 0.534, *p* < 0.01). Compression impulse was correlated with the left paddle impulse (r = 0.672, *p* < 0.01).

### 4.2. Comparative Analysis of the Best and Worst Performances

**Figure 9:**
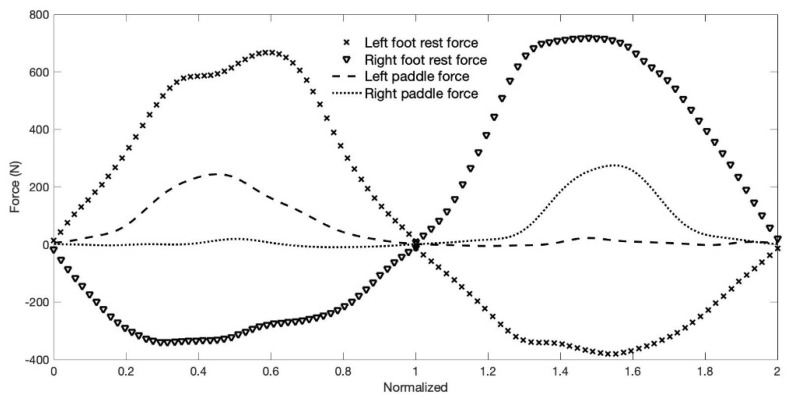
Mean force–time curves of the forces applied on the foot rest/strap and paddle of the kayaker with the best performance of the sample group. Data was time normalized and represents N = 238 strokes (119 complete stroke cycles).

**Figure 10:**
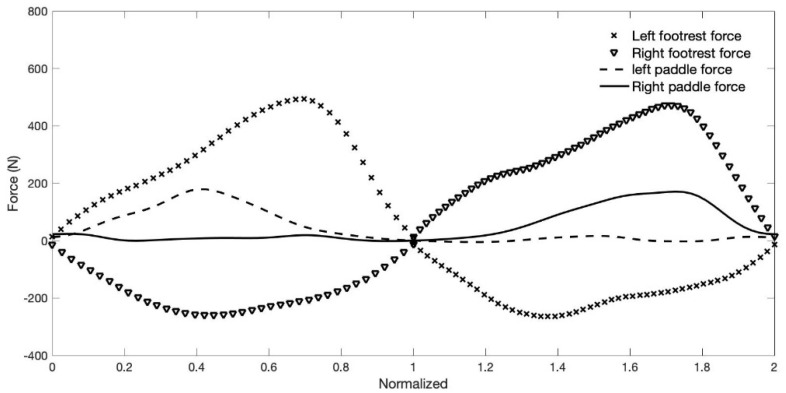
Mean force–time curves of the forces applied on the foot rest/strap and paddle of the kayaker with the worst performance of the sample group. Data was time normalized and represents N = 252 strokes (126 complete stroke cycles).

Mean power during the 2-min test was used as parameter to determine the best and worst performances (the difference in mean power between the two athletes was 88 W). When analyzing the mean force–time curves and results of the kayakers with the best (Figure 9) and the worst (Figure 10) performances, the results suggest differences, both in terms of magnitude and profile. 

The visual comparison of Figure 9 and Figure 10 suggests different ways and timings of force development. The best performance presents a tendency for curves with higher values, and in middle of the curve, a tendency for a plateau; the analyses also shows more pronounced slopes from and to zero force. The kayaker with the best performance reaches the peak forces on the paddle and foot rest from the same side and foot strap of the opposite side almost simultaneously, which is no longer observed in Figure 10, which presents the data of the athlete with the worst performance.

Table 4 presents the magnitude of the forces applied by the two kayakers, best and worst performances. Results show higher absolute values for the kayaker with the best performance, for each of the variables analyzed in terms of foot rest, foot strap, and paddle forces (peak and mean force, and impulse) when compared with the results from the kayaker with the worst performance. When the variables area presented was normalized to paddlers weight, the difference in % were lower and mainly in foot rest/strap force variables.

## 5. Discussion

This is the first study that combines paddle, foot rest and strap kinetic analysis in a group of elite kayakers. Previous studies have focused on the paddle or foot rest forces independently, however this issue has never been addressed in relation to performance [1,5,17]. Therefore, the main goal of the present study was to characterize the profile of the curves and magnitude of the forces exerted by the lower limbs of elite kayakers during 2 min on a kayak ergometer, simulating sprint race performance, synchronized with the forces applied on the paddle. Results show that in each stroke the forces applied on the foot rest and foot strap begun to be applied before the ones applied on the paddle shaft, as previously reported in a study testing only two kayakers (one male and one female) and performed on water [10].

Analyzing the magnitude of the forces, in terms of peak forces, the ones applied on foot rest (compression) doubled the ones applied on the paddle. Already, the forces on the foot strap (tension) were similar in magnitude to paddle forces. When analyzed in terms of mean values, the compression forces were more than three times and the tension forces almost two times, the forces applied on the paddle. The differences were higher when analyzed in terms of the mean values, compared with peak, due to the fact that in terms of curve shape, the forces applied on the foot rest and foot strap had a much higher tendency for a rectangular profile, instead of being closer to the triangular profile of the paddle forces. Another aspect to note was that there were no significant differences between left and right-side forces, for both the foot rest and foot strap as well as paddle, prevailing the idea of non-significant asymmetry in elite kayakers [17,18].

No direct and strong correlation was observed between the foot rest forces (magnitude and time-dependent variables) and the performance (power) as previously reported [19], except the correlation between the left compression force duration and the performance (r = 0.560, *p* < 0.05). If a longer compression force duration would be associated with a greater range of motion of the knee, results are in line with a previous study [3], that suggested that greater knee amplitude are associated with elite kayakers’ performance. Nilsson and colleagues [1] observed that kayak speed decreased by 16% with the restriction of movement of the lower limbs and also reported a high correlation between pushing forces and kayak velocity, which suggests that the force applied by the lower limbs considerably influences the kayaking performance [6]. Previously, Begon and colleagues [5], based on computer modeling to access the lower limb contribution to kayak performance, suggested that the legs produce 6% of the total propulsion on a sliding kayak ergometer. The correlation analysis of the compression and tension forces applied simultaneously (one side foot rest and the foot strap of the opposite side) led to the suggestion that the higher the compression force, the greater the tension force on the opposite side. Although this is the first study to show a positive linear correlation between the magnitude of the forces applied on the foot rest and foot strap, it is not possible, however, to define a cause–effect relation.

The analyses of the forces applied on the paddle showed that performance (mean power in kayak ergometer) correlated with paddle mean force (r = 0.792, *p* < 0.001) and paddle impulse (r = 0.701, *p* < 0.001). Was also observed a high correlation between these variables and kayak velocity [7], which is in line with the results of the present study. A significant correlation between performance and peak paddle force (lower correlation) was also presented, which is in line with the results of other paddle force profile studies in on-water situations [7]. Maintaining force values near the peak force throughout the water phase is more important than the peak power itself, thus generating a more effective paddle stroke and consequently propelling the kayak even more [2].

**Figure 11:**
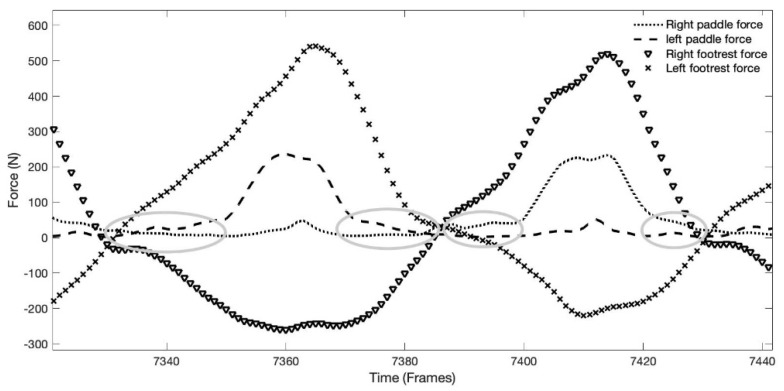
Raw data of the force–time curves of the forces applied on the foot rest/foot strap and paddle, showing the values close to zero before entry and after exit in water no the paddle force curve, identified with grey ellipses.

The paddle mean force/peak force ratio did not correlate with ergometer performance, however, previous studies concluded that the best performances were associated with higher values of this ratio [7,19]. Although the performance was correlated with the mean and impulse paddle forces and not with peak force, it suggests a tendency for a rectangular profile. However, this did not occur in the present study, probably as suggested by Begon and colleagues [19], due to the low forces values recorded before and after the supposed water entry/exit of the blade (Figure 11). These low values of tension on the kayak rope probably have interfered with the mean paddle force values and consequently with the mean force/peak force ratio. The low value, before and after the presumed delimitation of paddle stroke, was due to the recoil spring used in the ergometer, which continuously creates tension for collecting the cable. The use of a kayak ergometer instead of on-water performance was a limitation of the present study, however, it allowed to use an instrumented foot rest with a high number of load cells, although heavy, that collected compression and tension forces, which reflected the kayak force application by elite kayakers. Future studies should focus on the analysis in an on-water situation, collecting not only paddle and foot rest and strap forces, but also adding seat forces that is the other point of contact with the kayak.

The increase in paddle peak force was related to the increase of the compression peak force and impulse on the side of the stroke. Kayakers with higher paddle impulses also had higher compression impulses on the side of the stroke, suggesting that there is a dependence between the magnitude of the forces applied by the upper and lower limbs, on paddle and foot rest, respectively, as already had been previously suggested in a study in kayak ergometer [5] and lately confirmed in on-water situation [1]. Considering the correlations observed between the foot rest and strap forces with the forces applied on the paddle, and considering that these last were also correlated with performance, it suggests that, to a certain extent, the forces applied on the foot rest and strap are indirectly related to the performance. This is the first study to focus on the relation between the forces applied on paddle, foot rest, and strap, a to analyze it with respect to performance.

The analyses of the force–time curve shape showed that the forces of compression and tension (on opposite sides) began to be applied before forces being applied on the paddle, as also observed in a pilot study [9]. Therefore, each paddle stroke starts with lower limbs force development. Both forces applied on foot rest and strap also ended after the paddle force end, continuing until a certain moment of the aerial phase where both legs change from compression to tension and vice-versa, initiating the paddle stroke on the opposite side.

The compared analyses of the forces applied on the paddle, foot rest and foot strap, considering the best and worst performances (Figure 9 and Figure 10), showed differences in magnitude and shape of the force–time curves and consequently differences in performance. When considering the magnitude of the forces, results suggest that the greater magnitude can be associated with the best performance for both absolute and relative weight analyses of the forces. Another very interesting aspect was the timing in which the peak forces of compression, tension (on opposite sides) and on the paddle occurs (Figure 9 and Figure 10). For the kayaker with the best performance, the peak timing of the three forces tended to be simultaneous, unlike the kayaker with the worst performance. It was also observed in a previous study that international level kayakers showed closer peak synchronization compared to national elite kayakers [10]. However, it would be of interest to confirm the present results, since they suggest that better performances are dependent on the ability to develop higher forces, but also to be able to synchronize their peaks.

Performance in kayaking depends on the magnitude and the way forces are applied by the kayaker on the paddle and points of contact with the kayakers, but also the way this reduces drag forces as much as possible. Therefore, a higher magnitude of forces applied is not an exclusive condition that allows defining which will be the best performance, since it will have to be balanced with the drag forces that are being generated. In this way, kayakers with similar performances can have considerable differences in the magnitude of the forces applied, mainly to the foot rest and strap.

The comparison of the results of the kayakers with the best and worst performances showed that the one with the worst performance tended to take longer time to reach the peak force (low slope) of both left and right compressions and peak paddle force, which have been suggested as factors that limit the performance [7]. Already, the best performance showed a force curve shape where the peak forces were reached quickly and tended to remain for a period near the peak (plateau), which have been mentioned as a determinant of kayaking efficiency [1]. Also, the results in terms of ratios (mean force/peak force), for the best performance kayaker, showed a tendency for a more rectangular shape of the force–time curves [16].

The study presents unique data about the forces applied on the foot rest, strap, and paddle, correlating them with performance in kayak ergometer. It was shown that the forces applied on the foot rest and strap in each paddle stroke began to be applied earlier than the forces on the paddle and ended later, continuing till a certain moment of the aerial phase where both legs change from compression to tension and vice-versa, to initiate the paddle stroke on the opposite side. It was also observed that the higher the magnitude of the paddle forces, the better the performance. The paddle forces were found to be positively related to the magnitude of the forces applied on the foot rest, suggesting an indirect relation between the forces applied on the foot rest and the kayaking performance. When analyzing the shape of the force–time curves, the results suggest that best performances had a higher tendency for a rectangular shape, besides a tendency for a synchronized occurrence of the peak forces applied on the foot rest and paddle, on the same side, and on the foot-strap on the opposite side.

## 6. Conclusions

The analyses of the force–time curve, magnitude and shape, should be a prime objective in terms of technique analyses, not only the forces applied on the paddle but together with the forces applied by the kayaker on the foot rest and strap. The difference is that the force applied on the foot rest and strap is maintained continuously along a stroke cycle, changing each leg from compression to tension rapidly, while paddle force is applied intermittently, with periods of no force being applied. Scientists and coaches, regarding kayak technique and performance, should also focus on lower body performance, considering that the forces measured on the foot-rest doubled the ones applied on the paddle, besides the fact that the best performances had a higher magnitude of forces not only applied on paddle but also on the foot rest/strap. This study, as well as similar studies, have the potential to improve knowledge on the propulsion forces in kayaking, and consequently, help coaches and athletes improve sport performance. The deep knowledge about how elite kayakers apply forces (both magnitude and shape) and how the application of these forces are synchronized is also of interest. Understanding what the best kayakers are able to do, above the ones that are already in the elite group, can guide investigators to the precise aspects that influence performance.

## Figures and Tables

**Table 1:** Mean power (W) and mean stroke rate (stroke per minute) of each athlete and overall mean in the 2-min kayak ergometer performance test.

Kayaker	Mean Power (W)	Mean Stroke Rate (spm)
1	286	123
2	267	115
3	341	119
4	322	118
5	292	126
6	253	126
7	307	112
8	307	123
9	294	127
10	292	122
11	258	120
12	264	123
13	260	125
Mean ± SD	288 ± 26.9	121 ± 4.5

W, watt; spm, strokes per minute; SD, standard deviation.

**Table 2:** Variables related to the forces applied on foot rest (compression) and strap (tension) for left and right sides (mean ± SD). Positive values represent compression, and negative values represent tension forces.

Foot Rest/Strap Forces	Left	Right
	Mean ± SD	Mean ± SD
CPF (N)	543.27 ± 85.93	524.39 ± 88.36
TPF (N)	−240.09 ± 74.92	–231.05 ± 52.01
CMF (N)	283.04 ± 50.76	279.09 ± 64.13
TMF (N)	−152.72 ± 49.47	−147.74 ± 35.36
CI (N∙s)	143.62 ± 32.66	148.21 ± 31.89
TI (N∙s)	–72.77 ± 27.01	−65.94 ± 22.17
CFD (s)	0.52 ± 0.07	0.55 ± 0.05
TFD (s)	0.48 ± 0.06	0.45 ± 0.06
T.CPF (s)	0.32 ± 0.06	0.31 ± 0.05
T.TPF (s)	0.24 ± 0.08	0.21 ± 0.03
MF/PF RC (%)	52.31 ± 5.84	53.15 ± 5.68
MF/PF RT (%)	63.31 ± 4.29	63.92 ± 2.84

CPF, compression peak force; TPF, tension peak force; CMF, compression mean force; TMF, tension mean force; CI, compression impulse; TI, tension impulse; CFD, compression force duration; TFD, tension force duration; T.CPF, time to reach compression peak force; T.TPF, time to reach tension peak force; MF/PF RC, compression mean force/peak force ratio; MF/PF RT, tension mean force/peak force ratio; SD, standard deviation.

**Table 3:** Variables related to the forces applied on the paddle on the left and right sides.

Paddle Forces	Left	Right
	Mean ± SD	Mean ± SD
PPF (N)	236.37 ± 19.32	243.92 ± 28.89
PMF (N)	88.53 ± 6.87	88.68 ± 6.58
PI (N·s)	43.22 ± 4.76	42.23 ± 3.32
PFD (s)	0.49 ± 0.03	0.49 ± 0.04
T.PPF (s)	0.21 ± 0.02	0.21 ± 0.03
MF/PF RP (%)	38.82 ± 2.59	38.26 ± 5.27

PPF, paddle peak force; PMF, paddle mean force; PI, paddle impulse; PFD, paddle force duration; T.PPF, time to reach paddle peak force; MF/PF RP, paddle mean force/peak force ratio.

**Table 4:** The magnitude of the forces applied on the foot rest/foot strap and on the paddle by the two kayakers, with the best and worst performances. Absolute values and relative values to the kayakers’ weight, both presenting the differences in % between kayaker. Best performance n = 238 strokes and worst performance n = 252 strokes.

**Foot Rest/Strap Forces**	**Absolute Values**		**Dif. %**	**Foot Rest/Strap Forces**	**Relative to Weight**		**Dif. %**
	**Best**	**Worst**			**Best**	**Worst**	
lCPF (N)	676.78	501.56	30	lCPF (N·kg^−1^)	7.52	6.69	12
rCPF (N)	736.43	487.66	41	rCPF (N·kg^−1^)	8.18	6.50	23
lTPF (N)	−388.11	–267.14	37	lTPF (N·kg^−1^)	−4.31	−3.56	19
rTPF (N)	−345.16	–261.23	28	rTPF (N·kg^−1^)	−3.84	−3.48	10
lCMF (N)	387.57	272.47	35	lCMF (N·kg^−1^)	4.31	3.63	17
rCMF (N)	441.47	274.4	47	rCMF (N·kg^−1^)	4.91	3.66	29
lTMF (N)	–243.44	–167.33	37	lTMF (N·kg^−1^)	–2.70	−2.23	19
rTMF (N)	–230.99	−171.5	30	rTMF (N·kg^−1^)	–2.57	−2.29	12
lCI (N·s)	201.96	118.95	52	lCI (N·s·kg^−1^)	2.24	1.59	34
rCI (N·s)	209.02	154.26	30	rCI (N·s·kg^−1^)	2.32	2.06	12
lTI (N·s)	−114.46	–85.44	29	lTI (N·s·kg^−1^)	–1.27	−1.14	11
rTI (N·s)	−119.53	–66.09	58	rTI (N·s·kg^−1^)	–1.33	−0.88	41
**Paddle** **Paddle Forces**	**Absolute Values**		**Dif. %**	**Paddle**	**Relative to Weight**		**Dif. %**
	**Best**	**Worst**			**Best**	**Worst**	
lPPF (N)	273.4	225.3	19	lPPF (N·kg^−1^)	3.0	3.0	0
rPPF (N)	293.6	263.2	11	rPPF (N·kg^−1^)	3.3	3.5	−6
lPMF (N)	105.3	80.9	26	lPMF (N·kg^−1^)	1.2	1.1	9
rPMF (N)	99.1	88.9	11	rPMF (N·kg^−1^)	1.1	1.2	−9
lPI (N·s)	52.3	36.5	36	lPI (N·s·kg^−1^)	0.6	0.5	18
rPI (N·s)	47.0	39.4	18	rPI (N·s·kg^−1^)	0.5	0.5	0

Foot rest forces: lCPF, left compression peak force; rCPF, right compression peak force; lTPF, left tension peak force; rTPF, right tension peak force; lCMF, left compression mean force; rCMF, right compression mean force; lTMF, left tension mean force; rTMF, right tension mean force; lCI, left compression impulse; rCI, right compression impulse; lTI, left tension impulse; rTI, right tension impulse; paddle forces: lPPF, left paddle peak force; rPPF, right paddle peak force; lPMF, left paddle mean force; rPMF, right paddle mean force; lPI, left paddle impulse; rPI, right paddle impulse: Dif.%, difference in % between kayakers.

## Data Availability

The data presented in this study are available on request from the author.
